# A patient with ulcerated calcifying epithelioma of Malherbe in the pinna: case report

**DOI:** 10.1186/1758-3284-4-25

**Published:** 2012-05-21

**Authors:** Tahwinder Upile, Waseem Jerjes, Fabian Sipaul, Ann Sandison, Panagiotis Kafas, Mohammed Al-Khawalde, Hani Radhi

**Affiliations:** 1 Department of Head and Neck Surgery, Chase Farm & Barnet NHS Trust, Enfield, UK; 2 Head & Neck Unit, University College London Hospital, London, UK; 3 Department of Surgery, School of Dentistry, Al-Yarmouk University College, Baghdad, Iraq; 4 Oral and Maxillofacial Surgery Unit, AL-Mustansirya University's, Baghdad, Iraq; 5 UCL Department of Surgery, University College London, London, UK; 6 Leeds Institute of Molecular Medicine, Leeds, United Kingdom; 7 Department of Head & Neck Surgery, The Professorial Unit, The Royal National Throat, Nose and Ear Hospital, London, United Kingdom; 8 Department of Pathology, Charring Cross Hospital, London, UK; 9 Department of Oral Surgery and Radiology, School of Dentistry, Aristotle University, Thessalonica, Greece; 10 Oral and Maxillofacial Surgery Unit, Royal Medical Services, Amman, Jordan

## Abstract

**Introduction:**

Although pilomatrixomas are frequently encountered by dermatologists and pathologists in the differential diagnosis of head and neck lesions, this is not usually the case among head and neck surgeons.

A pilomatrixoma (calcifying epithelioma of Malherbe) is a benign tumour of the hair matrix cells. Histologically it is characterised by the presence of ghost cells, basophilic cells and foreign body cells. It may sometimes be difficult to histologically distinguish it from its malignant counterpart, the pilomatrix carcinoma.

We report an interesting case of an ulcerated pilomatrixoma of the pinna in a middle-aged Caucasian female.

**Case presentation:**

A 46-year-old Caucasian female presented with a one-month history of tender brownish lump on the pinna. Initially it was thought to represent a pyogenic granuloma. The lesion was treated by wide circular excision. Histopathological evaluation reported a benign calcifying epithelioma of Malherbe.

**Conclusion:**

A search of the world’s literature has led us to believe that this is a rare case of a calcifying epithelioma of Malherbe of the pinna. The rapid growth and ulcerative nature of this tumour makes this case even more unique.

## Introduction

Calcifying Epithelioma of Malherbe (CEM) or Pilomatrixoma was first described in 1880 by Malherbe and Chenantais as a benign tumour that occurs most commonly in children [1]. In 1949, Lever and Griesember suggested that it originates from the hair matrix cells [2]. It usually presents as a slow-growing lesion, but it can also grow rapidly and can be locally aggressive [3]. Its malignant counterpart, pilomatrix carcinoma or calcifying epitheliocarcinoma of Malherbe, is rare and was first described by Lopanski and Mihm in 1980 [4].

A calcifying epithelioma of Malherbe occurs most commonly in head and neck areas, especially cervical, frontal and temporal regions, eyelids and preauricular regions [5,6]; although the upper extremities, trunk and lower extremities can be affected, in decreasing order of frequency [7].

The peak incidence of pilomatrixoma is found between 8 and 13 years of age [8]. There is a slight female preponderance with a female to male ratio of 1.75:1 [9]. Clinically, it presents as a firm, irregular, reddish-blue, slow- growing, dermal or subcutaneous nodule that measures 0.5-5.0 cm in diameter [10,11]. It may be associated with a number of different conditions such as Gardner’s syndrome, myotonic muscular dystrophy, Rubinstein-Taybi syndrome, Turner’s syndrome, xeroderma pigmentosum and basal cell naevus syndrome [12-14].

## Case report

A 46-year-old Caucasian female presented with a one-month history of tender brownish lump on the pinna. The patient reported that the lump was rapidly increasing in size and its surface was ulcerating. The patient’s medical history was unremarkable.

**Figure 1:**
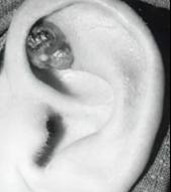
Clinical photograph of the pinna showing an ulcerated lesion.

Examination revealed a 1.4 by 1.0 cm tender, firm, non-fluctuant, ulcerated lesion; initially it was thought to represent a pyogenic granuloma (Figure 1). There was no palpable lymphadenopathy.

**Figure 2:**
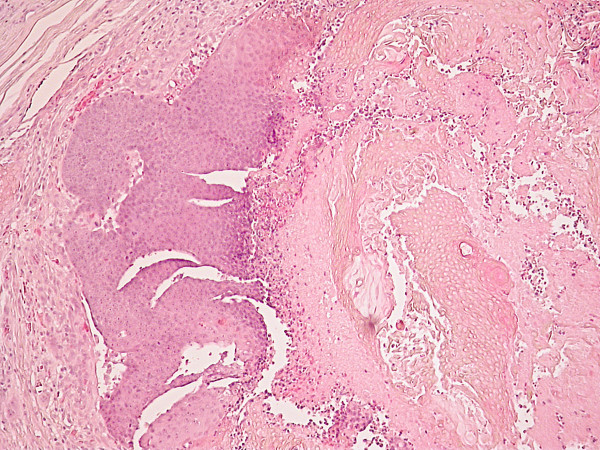
**H&E stained section taken with low-power objective.** This view shows the lobular architecture of the tumour and demonstrates the typical biphasic population of darkly staining basaloid cells (left) and the larger more eosinophilic ghost keratinocytes (right)

**Figure 3:**
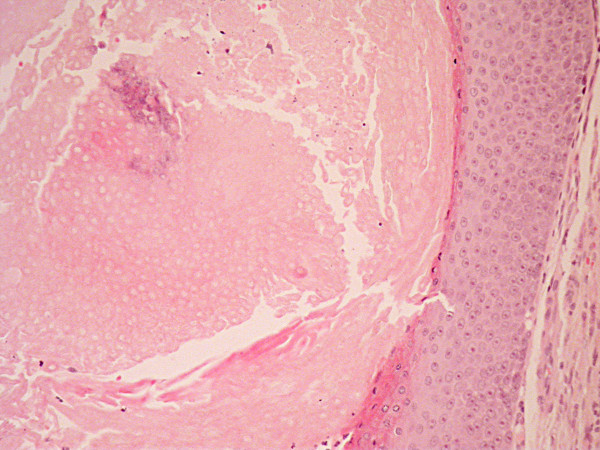
**H& E stained section taken with a medium power objective.** Squamous epithelium is shown on the right side adjacent to eosinophilic keratinous debris with visible ghost outline of tumour cells and calcification towards the centre. The granular layer is not in evidence and there is abrupt keratinisation in keeping with the predominantly pilar keratinisation seen in pilomatricoma.

**Figure 4:**
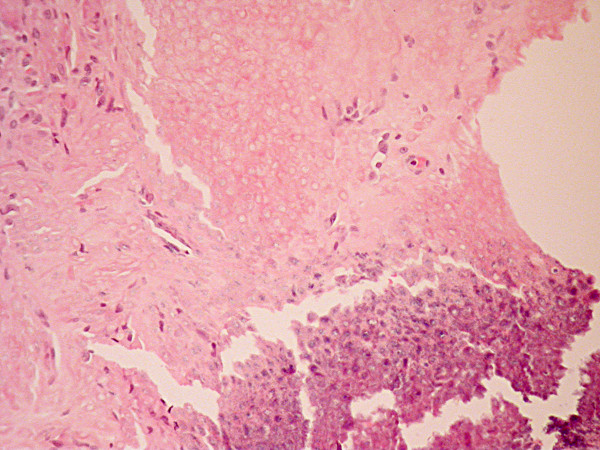
**H&E stained section taken with a medium power objective.** This shows maturation in pilomatricoma. The basaloid cells shown in the bottom right of the picture gradually become enlarged and more eosinophilic, the nuclei become pyknotic and eventually form the ghost cells characteristic of this tumour. There is foreign body reaction top left which is often seen in association with pilomatricoma

The lesion was treated by wide circular excision with a 4-6 mm margin followed by reconstruction of the defect with a split skin graft. Histopathological evaluation of the resected tissue reported a benign calcifying epithelioma of Malherbe (Figures 2, 3 and 4). The patient remains well after five years.

## Discussion

Calcifying epithelioma of Malherbe (pilomatrixoma) is a rare benign neoplasm of hair matrix cell origin [10]. It is one of the most common cutaneous tumours of skin appendages in patients less than 20 years [11]. To our knowledge, other cases have been described [15]. Other reported cases of a clinical variant called perforating pilomatrixoma, in which ulceration is typical [16].

The differential diagnosis for a head and neck pilomatrixoma includes a sebaceous cyst, ossifying haematoma, chondroma, degenerating fibroxanthoma, giant cell tumour, foreign body reaction, dermoid cyst, osteoma cutis, squamous cell carcinoma, basal cell carcinoma, amelanotic malignant melanoma and metastatic bone formation [6].

The typical histopathological features are the presence of mature, keratinised, karyolitic cells called ghost cells, basophilic cells, bony metaplasia, foreign body giant cell reaction [17] and less commonly pigmentation, transepidermal elimination and an infiltrative growth pattern [1]. The infiltrative growth pattern, the high mitotic rate and the presence of excessive basaloid cell proliferation characterize the aggressive type of pilomatrixoma, which may be histologically difficult to differentiate from basal cell carcinoma, proliferating pilar cyst and pilomatrix carcinoma [18]. It has the ability to locally invade adjacent structures and has the capacity to recur [19].

The malignant counterpart of pilomatrixoma is known as malignant pilomatrixoma, pilomatrix carcinoma or calcifying epitheliocarcinoma of Malherbe [4]. It is a rare finding, usually occurs in middle-aged patients and has a male to female ratio of 4:1 [20]. It has the tendency to recur locally [3].

Just above 50 cases have been described in the world’s literature. Of those cases, only five developed metastatic disease in the lungs, bones and viscera [3]. Pilomatrix carcinoma appears to be resistant to both primary chemotherapy and radiotherapy and therefore, radical surgery followed by postoperative radiotherapy has been advocated to ensure local control [3].

## Conclusion

A search of the world’s literature has led us to believe that this is a rare case of a calcifying epithelioma of Malherbe of the pinna. The ulcerative nature of this tumour makes this case even more unique, with only a few cases of ‘perforating pilomatrixoma’ reported so far of the pinna. The case was also of interest because of its rapid evolution with ulceration.

It should therefore be included in the differential diagnosis of any cutaneous lesion of the pinna, especially when considering lesions such as keratoacantomas, squamous cell carcinomas, basal cell carcinomas and amelanotic malignant melanomas, where treatment involves extensive resection followed by local or distant reconstructive techniques, and not just a simple excision.

## Consent

Written informed consent was obtained from the patient for publication of this case report and accompanying images. A copy of the written consent is available for review by the Editor-in-Chief of this journal.

## Competing interests

The authors declare that they have no competing interests.

## Authors’ contributions

TU, WJ, FS, AS, PK, MA, HR contributed to conception and design, carried out the literature research, manuscript preparation and manuscript review. All authors have read and approved the final version of the manuscript.
